# Integration policies shape ethnic-racial majorities’ threat reactions to increasing diversity

**DOI:** 10.1126/sciadv.adk8556

**Published:** 2024-05-29

**Authors:** Judit Kende, Dirk Jacobs, Eva G. T. Green, Linda R. Tropp, Yuen J. Huo, John F. Dovidio, Tomás R. Jiménez, Deborah J. Schildkraut, Olivier Klein

**Affiliations:** ^1^ Tilburg School of Social and Behavioral Sciences, Tilburg University, 5000 LE Tilburg, Netherlands.; ^2^ Institute of Psychology, Université libre de Bruxelles, 1050 Brussels, Belgium.; ^3^ Institute of Sociology, Université libre de Bruxelles, 1050 Brussels, Belgium.; ^4^ Institute of Psychology, University of Lausanne, 1015 Lausanne, Switzerland.; ^5^ Department of Psychological and Brain Sciences, University of Massachusetts Amherst, Amherst, MA 01003, USA.; ^6^ Department of Psychology, University of California, Los Angeles, CA 90095-1563, USA.; ^7^ Department of Psychology, Yale University, New Haven, CT 06520, USA.; ^8^ Department of Sociology, Stanford University, Stanford, CA 94305, USA.; ^9^ Department of Political Science, Tufts University, Medford, MA 02155, USA.

## Abstract

Increasing ethnic and racial diversity often fuels feelings of threat among ethnic-racial majorities (e.g., self-identified white Americans and European nationals). We contend that these threat perceptions depend on the policy context. Across four studies, we test whether more inclusive immigrant integration policies attenuate ethnic-racial majorities’ threat reactions. Studies 1 to 3 (*n* = 469, 733, and 1745, respectively) used experimental methods with white American participants in the United States. Study 4 (*n* = 499,075) used secondary analysis of survey data comparing attitudes of nationals in 30 European countries and measured the impact of actual changes in diversity and policies over 10 years. Our results show that integration policies shape threat reactions even in those situations when increasing diversity could be seen as the most threatening: when narratives highlight the majority’s impending minority position or when diversity suddenly increases. When policies are more inclusive toward immigrants, ethnic-racial majority participants report less threat (or no threat) in response to increasing diversity.

## Teaser

When policies are more inclusive toward immigrants, increasing diversity feels less or not at all threatening to majority members.

## INTRODUCTION

Because of growing migration, declining fertility rates, and population aging, Western societies are becoming more ethnically and racially diverse than ever before. Public narratives in many Western countries present increasing diversity as a problem that would be inherently threatening to ethnic-racial majorities and spark their concern about demographic changes in their societies (*1*–*3*). Ethnic-racial majorities could feel threatened when the size of their group diminishes relative to the presence of ethnic-racial minorities, because they may fear losing their political, social, economic, and/or cultural power and status (*4*–*6*). Correspondingly, when majorities see growing diversity as a threat, they express more negative emotions and attitudes toward ethnic-racial minority groups, engage in more discrimination against minority groups, and are more likely to support policies that restrict minority rights (*5*, *7*). In this paper, we use “ethnic-racial diversity” to refer to the population of immigrants and to nonimmigrant ethnic and racial minority groups.

We argue that ethnic-racial majorities’ threat perceptions depend on the local (state or country level) policy context because policies either normalize and equalize or problematize diversity. Across four studies, we test whether more inclusive policies attenuate ethnic-racial majorities’ threat responses to increasing diversity. We use survey-based experiments (studies 1 to 3) and a longitudinal survey (study 4) to offer causal evidence regarding the mitigating role of inclusive policies on ethnic-racial majorities’ threat responses.

To date, empirical studies show mixed results regarding the potential effects of increasing diversity on perceived threat (*8*). Of particular relevance, a recent meta-analysis indicates that most studies show no significant link between increasing ethnic-racial diversity and ethnic-racial majorities’ threat perceptions, but about one-fourth of the papers documented that growing diversity is associated with greater feelings of threat (*8*). Recent studies highlight two conditions under which increasing diversity could be seen as especially threatening. The first condition involves the narratives that frame these demographic shifts. A robust line of experimental research shows how narratives highlighting that in the future, ethnic-racial majorities will become numerical minorities provoke threat reactions (*7*, *9*, *10*). The second condition involves rapid growth in ethnic-racial diversity, bolstered by converging evidence from three large-scale multinational surveys (*11*–*13*). Together, it appears that there are critical conditions, such as prevailing narratives about losing one’s majority position or rapid increases in diversity, that are especially likely to provoke threat reactions among ethnic-racial majorities.

Could inclusive policies reduce threat reactions among ethnic-racial majorities under these critical conditions? Previous research has shown that actual inclusive immigrant integration policies are related to lower threat perceptions among native-born Europeans (*14*, *15*). Similarly, an experiment shows that discourse about more inclusive immigrant integration policies induces positive emotions among self-identified white Americans (*16*). Furthermore, anti-immigration attitudes also tend to be lower in sociopolitical contexts with inclusive policies and high immigrant presence (*17*). However, prior research has not yet tested whether inclusive immigrant integration policies mitigate ethnic-racial majorities’ threat reactions under exacerbating conditions, such as narratives highlighting that the ethnic-racial majority will lose its majority position or during rapid increases in ethnic-racial diversity. In addition, given that prior studies have drawn largely on cross-sectional analyses, they cannot provide clear support for the causal role of policies..

Two explanations have been offered regarding why inclusive policies may attenuate ethnic-racial majorities’ threat reactions (*14*–*17*). First, inclusive policies normalize diversity, conveying that policymakers and influential others are supportive of immigration, thereby contributing to more inclusive social norms (*18*, *19*). Inclusive norms could reframe how people interpret and respond to narratives highlighting shifts in numerical status or to rapid increases in diversity. Thanks to such normative reframing, individuals may view demographic changes as harmless or even beneficial. Second, inclusive policies equalize groups in diverse contexts, allowing immigrants to improve their societal positions (*20*, *21*). For example, during times of rapid demographic changes, inclusive policies facilitate the labor market integration of newcomers, improving their socioeconomic positions (*22*, *23*). Greater social, economic, and political integration may allow for more frequent and equal status encounters between ethnic-racial majority and minority group members and more favorable public representations of minority groups, and these encounters and representations can also diffuse threat perceptions (*24*–*26*). We therefore propose that inclusive immigrant integration policies attenuate majorities’ threat reactions even under critical conditions such as when narratives about their impending minority position prevail and when diversity rapidly rises.

### The present research

We conducted four studies to examine whether inclusive local (national or regional (state) integration policies mitigate the threat-inducing effects of increasing diversity among ethnic-racial majorities. We used survey experiments and longitudinal multilevel surveys, allowing us to draw causal conclusions about the unique and interactive effects of increasing diversity and inclusive policies. In each study, we either exposed participants to a narrative frame about rising ethnic-racial diversity or tested the effect of actual rapid growth in diversity. When exposing participants to a narrative about increasing ethnic-racial diversity, we highlighted how the ethnic-racial majority group would lose its numerical majority status position, because this framing has been shown in other research to be particularly threatening (*27*).

We conducted the research in the context of debates about rising ethnic-racial diversity in the United States and Europe and focused on the facets of ethnic-racial diversity highlighted in political and scientific debates (*28*, *29*). In the US, the largest ethnic-racial minority groups, i.e., Hispanic/Latinx, Black, and Asian Americans, have constituted part of American society over generations, and they are commonly racialized in public and academic discourse and everyday life (*3*, *30*). Moreover, especially nonwhite immigrants are also often racialized (*31*, *32*). In Europe, major ethnic-racial minority groups are immigrants and descendants of immigrants who arrived in Europe largely in the past 60 years (*33*). In the European context, their minority status is commonly tied to markers of ethnic origin (such as language, religion or heritage). While group differences usually refer to ethnic origin, these markers are regularly racialized in public representations of nonwhite immigrants in particular (*29*, *34*). Therefore, in this paper, we use “ethnic-racial diversity” to refer to diversity in relation to immigrant and nonimmigrant ethnic-racial minority groups across the American and European contexts.

Studies 1, 2, and 3 were implemented in the United States using experimental methods, taking into account ethnic-racial diversity linked to immigration and diversity borne from longer-standing presence of minority groups in the US. We complemented the experimental data with regional data on diversity and policies across US states. This combination enabled us to test whether experimental exposure to a potentially threatening narrative about increasing diversity would vary across US states with more inclusive (or less inclusive) immigration policies. Furthermore, we examined the effect of both policy discourses and actual policies, as discourses and policies often diverge (*35*). Study 4 was conducted in Europe, focusing on growing ethnic-racial diversity in relation to immigration and drawing on representative longitudinal survey data comparing 30 European countries over 10 years. Crucially, our European study included data from 2015, 2016, and 2017 when the number of immigrants arriving in European countries reached record numbers (*36*, *37*). In each study, we focused our analysis on the self-identified ethnic-racial majority respondents within each national context. In line with theoretical approaches and empirical work on the downstream consequences of threat perceptions, our dependent variables across studies were threat perceptions, emotions, and attitudes toward immigrants and immigration policy preferences (*10*, *38*). We implemented multilevel modeling in all four studies. Table 1 provides an overview of the four studies.

**Table 1.** Overview of the four studies.

Study	Assessment of increases in ethnic-racial diversity	Assessment of inclusive policies	Context
1	Narrative about increasing diversity (manipulated)	Inclusiveness of actual policies (measured)	US states
2	Actual ethnic-racial diversity (measured)	Discourse about changing policy (manipulated)	US states
3	Narrative about increasing diversity (manipulated)	Discourse about changing policy (manipulated) and inclusiveness of actual policies (measured)	US states
4	Change in actual ethnic-racial diversity over time (measured)	Change in inclusiveness of actual policies over time (measured)	European countries

### Overview of study goals and hypotheses

First, we expected to replicate the threat effect under critical conditions across all four studies. Specifically, we expected that in studies 1 and 3, experimental exposure to a narrative about white people becoming a minority in the US would induce threat reactions among white Americans. In study 4, we expected that rapid increases in immigrant inflow would lead to greater threat reactions among nationals across European countries. Second, we expected that ethnic-racial majorities’ threat reactions would be lower in places with more inclusive local policies. We expected similar patterns of effects when actual state or country immigrant integration policies were measured in studies 1, 3, and 4, and when policy discourses were manipulated experimentally in studies 2 and 3. Third, we expected that more (versus less) inclusive local policies would attenuate majorities’ threat reactions under critical conditions of exposure to narratives about increasing diversity and rapidly growing actual ethnic-racial diversity. Here, we expected to find similar effects when narratives about increasing diversity were experimentally manipulated and when we measured actual demographic changes over time.

## RESULTS

### Study 1

In study 1, our aim was to test whether potential threat reactions to a narrative frame about increasing diversity would be attenuated in US states with more (versus less) inclusive policies for immigrant integration. We used secondary data from a study that experimentally exposed white Americans to a vignette describing how due to growing ethnic-racial diversity, “white people are becoming a minority” (*39*). We compared this condition that discussed “white Americans becoming a minority” to a control condition (the study included further experimental conditions unrelated to the present study). We analyzed the effects of these conditions on positive and negative emotions as outcomes in separate models. We selected this study by Levy and Myers for secondary analysis because we could identify the participants’ state of residence and test the hypothesized cross-level interaction between the individual-level effect of the experimental manipulation and state-level policies. For the purposes of the present research, we tested whether the “increasing diversity” effect varies depending on the inclusiveness of the actual immigrant integration policies of the participants’ state of residence (*40*). The final sample included 469 non-Hispanic whites who were either in the increasing diversity or in the control condition, indicated their state of residence, and passed the attention check. See the Supplementary Materials for detailed information on the materials and methods (text S1 and tables S1 and S2).

**Fig. 1.**
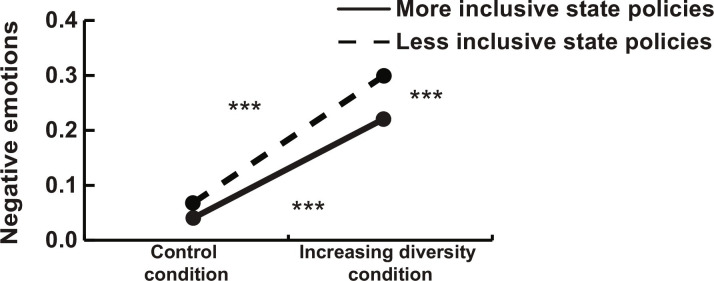
Study 1. Increasing diversity experimental manipulation (dichotomous variable) and actual state immigrant integration policies (continuous variable) predicting negative emotions. Less inclusive policies defined as −1 SD from the mean and more inclusive policies defined as +1 SD from the mean.***P < 0.001 (two-tailed).

The first set of models focused on negative emotions as the outcome variable. In line with our expectations, the increasing diversity condition induced more negative emotions (*b* = 0.202, SE = 0.024, and *P* < 0.001; see models in table S6). Please note that we report unstandardized coefficients throughout the paper, because standardizing the coefficients would distort the interaction effects in multilevel models (*35*). Then, we added age, gender, education, and political partisanship as control variables. As Fig. 1 shows, more inclusive state integration policies were positively related to less negative emotions (*b* = −0.160, SE = 0.044, and *P* < 0.001). The experimental effect was moderated by the actual state immigrant integration policies (*b* = 0.166, SE = 0.065, and *P* = 0.010). As Fig. 1 shows, in line with our hypothesis, the tendency for increasing diversity to provoke negative emotions was attenuated among white participants who lived in states with more inclusive immigration policies (Fig. 1). More specifically, the difference between less and more inclusive states translates into a 9% difference in negative emotions (percentage of the full range of the scale).

Please note that throughout the paper, when we plot the interactions, we calculate the simple effects in the interaction models at high and low values of the moderator or plotting experimental and control conditions, see specific plots for more information. In studies 1, 2, and 4 the independent variables are plotted with lines because they represent continuous variables. In study 3, we plot interactions between dichotomous independent variables using bar graphs.

**Fig. 2.**
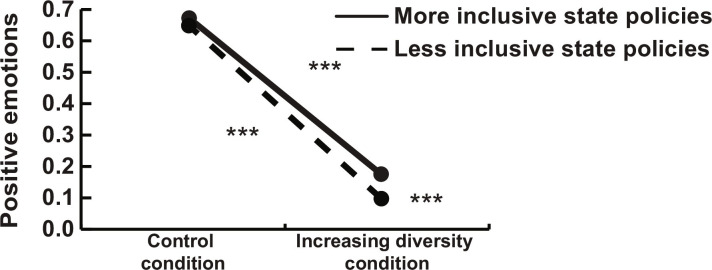
Study 1. Increasing diversity experimental manipulation (dichotomous variable) and actual state immigrant integration policies (continuous variable) predicting positive emotions. Less inclusive policies defined as −1 SD from the mean and more inclusive policies defined as +1 SD from the mean.***P < 0.001 (two-tailed).

A second set of models uses positive emotions as the outcome. Consistent with our expectations, we found that the increasing diversity condition induced less positive emotions (*b* = −0.530 SE (0.027), and *P* < 0.001; see models in table S7). We found that more inclusive state integration policies were related to more positive emotions (*b* = 0.144, SE = 0.058, and *P* = 0.013). Paralleling the analysis with negative emotions, the interaction model showed that the experimental effect was moderated by immigrant integration policies (*b* = 0.173, SE = 0.048, and *P* < 0.001). The plotted interaction in Fig. 2 shows that, in line with our hypothesis, the tendency for increasing diversity to induce less positive emotions was attenuated among white participants who lived in states with more inclusive immigration policies. Similar to the analysis with negative emotions, the difference between less and more inclusive states corresponds to 9% difference in positive emotions (percentage of the full range of the scale).

As robustness checks, we tested the possibility that these results are due to other plausible context-level factors, such as the political partisanship of state leaders and whether states lie in the US South or on the southern or northern US border. Specifically, we tested the effects of the experimental manipulation and state immigrant integration policies interaction on both negative and positive emotions controlling for the percentage of seats held by Democrats in the state House and Senate and including a dummy variable for Southern states and for border states (table S8). The diversity manipulation by immigrant integration policy interactions remained significant for both negative emotions (*P* = 0.012, *P* = 0.015, and *P* = 0.010 when including % Democratic seats, dummy for Southern, and border states, respectively) and positive emotions (*P* < 0.001 in all three robustness checks), giving us greater confidence in the causal effect of policies.

### Study 2

In study 2, we examined whether an experimental manipulation describing an inclusive policy would minimize threat reactions, especially at high levels of diversity (understood here as a higher relative presence of immigrants). We reanalyzed data from Huo *et al.* (*16*) who experimentally investigated the effect of a policy discourse describing proposed welcoming or hostile immigrant integration policies. The experiment includes a representative sample of residents from two US states, Arizona and New Mexico. While both Arizona and New Mexico lie on the US-Mexico border, immigrant presence varies greatly across counties within each state. We exploited this variation in immigrant presence and tested whether the policy manipulation would be especially consequential in counties with high levels of ethnic-racial diversity. The final sample included 733 non-Hispanic white participants. We tested the effect of integration policies on positive emotions. See the Supplementary Materials for detailed information on the materials and methods (text S2 and table S3).

**Fig. 3.**
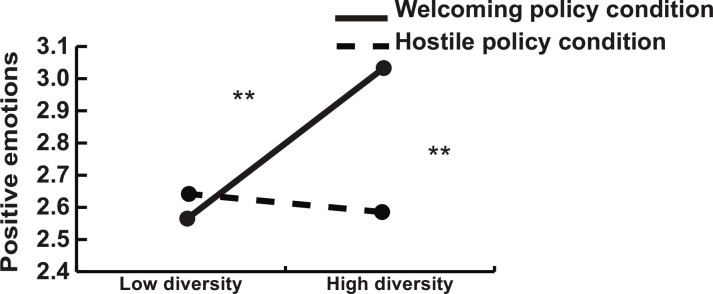
Study 2. Actual county diversity (continuous variable) and policy manipulation (dichotomous variable) predicting positive emotions. Low diversity defined as −1 SD from the mean and high diversity defined as +1 SD from the mean.**P < 0.01 (two-tailed).

As a first step, we replicated the effect of policy conditions on emotions from the original study by Huo *et al*. (*16*). We found that the more hostile policy condition predicted less positive emotions (*b* = −0.352, SE = 0.040, and *P* < 0.001). Then, we added age, gender, education, political partisanship, and proportion of immigrants in the county as control variables (see table S9 for models). To test our hypothesis, we added a cross-level interaction between the individual-level policy condition and the county-level immigrant percentage. The interaction term was marginally significant (*b* = −4.429, SE = 2.462, and *P* = 0.072). Figure 3 shows that, in line with our expectations, welcoming policies induced more positive emotions compared to hostile policies, yet this participant-level effect is observable only among participants in counties with high levels of diversity. More precisely, there was a 30% difference in positive emotions between the less and more diverse counties (percentage of the full range of the scale).

### Study 3

In study 3, we tested whether a welcoming policy manipulation would attenuate the potentially threatening effect of a narrative about increasing diversity, namely, a narrative about white people becoming a numerical minority in the US. We also tested for a three-way interaction involving the increasing diversity experimental manipulation, the experimental policy manipulation, and actual state policies. Here, we examined whether the policy discourse and increasing diversity interaction would depend on the actual immigrant integration policies in US states; we did not put forward a hypothesis regarding the possible direction of effects for the three-way interaction. See the Supplementary Materials for detailed information on Materials and Methods (text S3 and table S4).

The experimental materials for study 3 were based on those used in previous studies (see the Supplementary Materials for verbatim stimuli) (*9*, *16*). We used the same measures of state immigrant integration policies in study 1 (*31*). We sampled 1745 white Americans in an online survey using Prolific. The sample size was based on a priori power analysis (see link to preregistration in text S3). We included five outcome variables commonly used in experimental studies on the topic: racial status threat, negative emotions, immigration threat perceptions, immigration policy support, and attitudes toward undocumented immigrants (see the Supplementary Materials for items). We controlled for age, gender, education, and political partisanship in the analysis. We prioritized outcome variables related to immigration because our policy manipulation and our hypothesized moderator of state policies both concerned immigrants.

First, in partial support for our hypothesis, we found that the increasing diversity narrative was related to higher racial status threat and more negative emotions; however, increasing diversity did not have a statistically significant effect on the other three outcome variables (see tables S10 to S14 for models). We also tested the interactions with participants’ political partisanship. The increasing diversity effect was moderated by partisanship on negative emotions but not the other outcomes. When looking at the interaction with negative emotions, more politically conservative participants reported more negative emotions in response to the increasing diversity manipulation, but more liberal participants did not. Second, in contrast with our expectations, the inclusive policy manipulation did not induce more positive responses on any of the outcome variables (no significant main effects). We did not find any significant interactions with participants’ political partisanship. However, more inclusive state immigrant integration policies were related to more negative emotions (*b* = 0.433, SE = 0.216, and *P* = 0.045) and less restrictive immigration policy preferences (*b* = −0.347, SE = 0.198, and *P* = 0.080), although we did not find significant effects on the other outcomes. Third, we added the interaction between the increasing diversity condition and the policy condition to test whether the more inclusive policy condition would attenuate the threat effect of the increasing diversity discourse on either racial status threat or negative emotions. The interaction effect was not significant on either racial status threat (*b* = −0.094, SE = 0.080, and *P* = 0.236) or negative emotions (*b* = −0.156, SE = 0.086, and *P* = 0.068).

Lastly, we explored whether the interaction between the increasing diversity condition and the policy condition would be further moderated by the actual state immigrant integration policies by adding a three-way cross-level interaction among these three terms. We found a significant effect for the interaction on racial status threat, negative emotions, immigration threat perceptions, and immigration policy support (but not on attitudes toward undocumented immigrants). We plotted these interactions between the increasing diversity condition and the policy conditions separately in states with more inclusive and more exclusive policies.

**Fig. 4.**
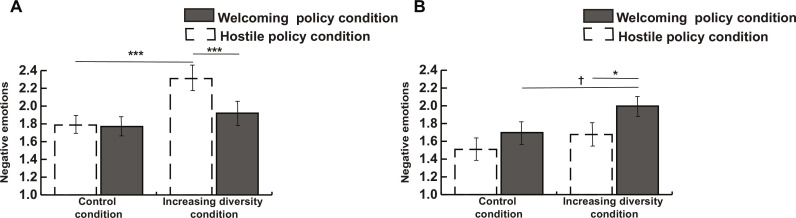
Study 3. (**A**) Increasing diversity manipulation (dichotomous variable) and policy manipulation (dichotomous variable) predicting negative emotions in states with more inclusive policies (continuous variable, defined as +2 SD from the mean). (**B**) Increasing diversity manipulation and policy manipulation predicting negative emotions in states with less inclusive policies (defined as −2 SD from the mean). To enhance readability of the figures, in study 3 we plotted the interactions defining inclusive and exclusive policies as ±2 SD from the mean. For exclusive policies (B), this value is theoretical, and no states had such exclusive policies. Error bars represent SEs. *P < 0.05, ***P < 0.001 (two-tailed).

We first describe the results concerning the states with more inclusive policies. Figure 4A shows the two-way interaction on negative emotions in more inclusive states. It shows that in states with more inclusive policies, when participants were told that policies are becoming more welcoming, the increasing diversity condition was not related to more negative emotions. This finding also replicated on the other outcome measures (see figs. S1 to S6). In contrast, when participants were told that policies are becoming more hostile, the increasing diversity condition induced more negative emotions. More precisely, there was a 6% difference in negative emotions between the hostile and welcoming policy manipulation (percentage of the full range of the scale). These results replicated on some of the other outcome variables such as perceptions of racial status threat, and support for restrictive immigration policies, but not on perceptions of immigration threat.

Turning to the states with more exclusionary policies, when participants were told that policies are becoming more welcoming, the increasing diversity condition consistently resulted in worse outcomes across all the outcome variables, see, for example, Figure 4B plotting the interaction on negative emotions. To the contrary, when participants were told that policies are becoming more hostile, the increasing diversity condition did not exacerbate any of the outcomes. There was a 5% difference in negative emotions between the hostile and welcoming policy manipulation (percentage of the full range of the scale). See figs. S1 to S6 for other outcomes.

To test the robustness of the effects, we also conducted the analyses controlling for the percentage of seats held by Democrats in the state House and Senate (table S15). These analyses revealed significant three-way interactions for racial status threat (*P* = 0.038) and immigration threat perceptions (*P* = 0.042), and marginally significant interactions for negative emotions (*P* = 0.060), and immigration policy (*P* = 0.055). Note that as we report three-level cross-level interactions in study 3, adding two context-level controls (percentage of white population and the percentage of seats held by Democrats) places extremely high demands on the models (*41*). Thus, these results give us greater confidence in the causal effect of policies.

### Study 4

Last, in study 4 we examined whether actual inclusive immigrant integration policies attenuate the potential threat reactions to increasing immigration. We drew on the largest and most conclusive dataset on immigration attitudes of which we are aware (*13*). The dataset in our study comprised the aggregated opinion on immigration of 499,075 participants gathered in 385 nationally representative surveys in six survey projects across 30 European countries. The full dataset is larger, we only used the data that we could match to the policy scores. The dataset drew on data from commonly used survey projects such as the European Social Survey and the World Values Survey, but unlike most survey projects (e.g., the European Social Survey), it provided data for each year. It covered the period between 2007 and 2017 with yearly data on immigrant inflow (i.e., immigrants arriving in each country), on policies and on attitudes. To measure policies, we used the Migrant Integration Policy Index, the most comprehensive and detailed index of immigrant integration policies in Europe (*42*). We simultaneously tested the impact of the average immigrant inflow and policies in a given country over this 10-year period and the impact of yearly changes in immigrant inflow and policies in each country. In other words, when we tested the impact of yearly changes, we measured how far the inflow and the policies differed in a given year from the average inflow or policies in that country. This 10-year period includes the years 2015, 2016, and 2017, when the number of immigrants arriving in European countries reached record numbers. Therefore, the data are especially suitable for investigating the impact of rapid increase in diversity by looking at the changes in yearly inflow. See the Supplementary Materials for detailed information on the materials and methods (text S4 and table S5).

**Fig. 5.**
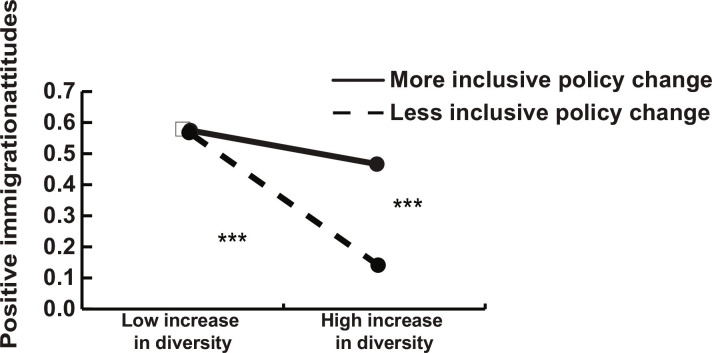
Study 4. Change in diversity (continuous variable) and change in policy (continuous variable) predicting positive immigration attitudes (low increase in diversity and less inclusive policy change defined as −1 SD from the mean, high increase in diversity, and more inclusive policy change defined as +1 SD from the mean).***P < 0.001 (two-tailed).

In a first step, we examined the decomposition in the variance and found that the variance is significant at both the within-country (*b* = 0.203, SE = 0.030, and *P* < 0.001) and between-country levels (*b* = 1.342, SE = 0.296, and *P* < 0.001), indicating that there is meaningful variation in immigration attitudes within countries over the years and also across countries. We also probed the variance in immigrant inflow and policies and found that similarly, there is meaningful variation both at the within and the between country level: inflow within (*b* = 0.092, SE = 0.027, and *P* = 0.001), inflow between (*b* = 0.863, SE = 0.134, and *P* < 0.001), policies within (*b* = 7.438, SE = 1.947, and *P* < 0.001), and policies between (*b* = 186.286, SE = 49.300, and *P* < 0.001). Moreover, we found that higher mean immigrant inflow in a country was related to more positive immigration attitudes (*b* = 0.794, SE = 0.266, and *P* = 0.003), whereas higher yearly changes in immigrant inflow in a country predicted more negative attitudes (*b* = −0.422, SE = 0.121, and *P* < 0.001). In addition, when immigrant integration policies are more inclusive in a country, immigration attitudes are more positive (*b* = 0.040, SE = 0.009, and *P* < 0.001) and yearly changes toward more inclusive policies also improve attitudes (although the relation was only marginally significant; *b* = 0.031, SE = 0.019, and *P* = 0.097). The interaction between yearly change in immigrant inflow and yearly changes in policies was significant (*b* = 0.101, SE 0.030, and *P* = 0.001). Plotting the interaction showed that in the case of a high yearly increase in immigrant inflow, attitudes worsen when policies become less inclusive (Fig. 5). In contrast, even when the immigrant inflow increases sharply in a given year, adopting more inclusive policies improves attitudes in line with our expectations. The difference between countries with more or less inclusive policy change corresponds to a 6% difference in attitudes (percentage of the full range of the scale). We replicated these findings over the main effect of other country-level factors that could potentially exacerbate attitudes, such as national wealth, unemployment rates, income inequality, proportion of immigrants, and proportion of far-right seats in parliament to provide further evidence for the causal role of policies. We also tested a reverse causation model with attitudes predicting policies. In support of our argument that policy change influences attitudes, we found that attitudes predict the average policy in a country, but not the yearly policy changes. See tables S16 to S18 for models.

## DISCUSSION

Ethnic and racial diversity is increasing in most Western societies. Many politicians and social scientists claim that threat reactions are inevitable and public narratives often highlight the downsides of growing diversity (*1*, *7*, *8*). Yet, members of ethnic-racial majority groups can react to rising diversity by feeling threatened or by embracing the changes. Across four studies, we investigated whether inclusive immigrant integration policies mitigate the potential threat reactions of majority members. In three studies, we used experimental methods and probed how the interplay between increasing diversity and policies shapes white Americans’ threat reactions in the United States. In study 4, we used longitudinal survey analysis to examine how increasing diversity and changing policies jointly affect majority nationals’ responses over a 10-year period in 30 European countries. We used these methods to be able to draw causal conclusions about the effects of increasing diversity and inclusive policies, going beyond previous largely cross-sectional integration policy analysis.

In line with previous studies, we found that two critical conditions fueled threat reactions: narratives about the impending minority position of the ethnic-racial majority group and rapid growth in diversity (*6*, *7*, *11*–*13*). We also found that even under these critical conditions, inclusive immigrant integration policies attenuated or completely buffered ethnic-racial majorities’ threat reactions. We argue that majorities’ threat perceptions depend on the local (state or country level) policy context because inclusive policies normalize diversity and equalize groups in diverse contexts.

While the policy effects were overall consistent, we uncovered intriguing differences between the effects of policy discourses (in the form of experimental vignettes) and the effects of actual immigrant integration policies. On the one hand, we consistently found that actual immigrant integration policies mitigated threat reactions. More specifically, study 1 showed that inclusive state immigrant integration policies attenuated threat effects in the US context. Similarly, study 4 demonstrated that changes toward more inclusive country immigrant integration policies buffered potential threat reactions when diversity increased sharply in the European context. On the other hand, the effect of discourses about inclusive policy changes (in the form of vignettes) was more mixed. The inclusive policy vignette contributed to attenuated threat responses in more diverse US counties (marginally) in study 2, and it attenuated the threat effect in study 3. However, study 3 also revealed that the effect of the policy vignette was conditional on the actual state policies.

We speculate that these differences stem from actual policies and policy discourses operating through different mechanisms. Actual inclusive policies both equalize and normalize diversity. Thus, actual inclusive policies shape threat perceptions by extending equal rights to immigrants and by communicating an inclusive norm (*14*–*16*, *20*). First, by granting more equal rights, actual policies equalize the societal position of immigrants and thus enable more frequent and egalitarian encounters with them when diversity grows (*20*, *24*). Crucially, majority members need not be aware of these policies for the policies to affect their encounters with immigrants. For example, more inclusive policies allow immigrants to work in higher-status occupations (*43*). Therefore, majority members are more likely to encounter immigrants as their colleagues or their neighbors. Such frequent and egalitarian encounters could reduce ethnic-racial majority members’ threat perceptions even when narratives highlight the majority groups’ impeding minority position or when ethnic-racial diversity is rapidly increasing (*25*, *44*). Second, actual inclusive policies normalize diversity by signaling that policymakers are supportive of immigration and immigrants, and such norms can also diffuse perceptions of threat (*18*, *45*). In contrast with actual policies, policy discourses create a normative effect, but they do not equalize the rights or position of immigrants. These different mechanisms might explain why the effect of policy discourses is more mixed than the effect of actual policies. Majority members might support or resist such inclusive norms communicated by policy discourses and thus be more or less impacted by the normative effect of an inclusive policy discourse (*16*, *46*). To the contrary, even if majority members are less supportive of diversity, their perceptions would be affected by more egalitarian and frequent contact (*47*).

Despite the large sample size, we found overall small and inconsistent effects of the increasing diversity manipulation in study 3. These findings are incongruent with the primary studies and also with a large body of literature on the subject (*7*, *10*). At the same time, our findings align with recent studies showing no effect of growing diversity on threat using the same experimental paradigm, or a threat effect only on racial status threat and emotions, but not on attitudes or policy preferences (*39*, *48*, *49*). One potential explanation is that, in the US context, white Americans may have become more habituated to discourse about increasing diversity. The Census Bureau’s projection about the United States becoming a majority-minority country was published 15 years ago at the time of writing and has been a part of the political and social discourse ever since. Similar to how ethnic-racial majority members habituate to actual rising diversity over time (*11*), they might also acclimate to the idea of becoming a numerical minority before the full demographic changes take place.

We base our conclusions on analyses of different relevant data sets using multiple methods. Still, our study was limited by the availability of indices on ethnic-racial equality. Our explanatory focus is on increasing ethnic-racial diversity from the perspective of the majority group who might lose their numerical majority position. This growth in diversity is partly driven by immigration and partly by the growing proportion of minority groups in society. The immigrant integration policies we highlighted as equalizing and normalizing forces first and foremost regulate the rights of immigrants (*20*). In contrast, the policies only pertain to a lesser degree to minority members of immigrant origin. Furthermore, they do not affect the societal status or position of historical ethnic-racial minority groups who at least formally have equal rights. Thus, our measure of equal rights for immigrants captures only one facet of inequality in society. At the same time, precisely because immigrant integration policies only affect one aspect of inequality, these findings provide evidence that equalizing and normalizing diversity are effective ways to reduce threat.

Our findings have clear theoretical implications for perspectives on conflict and threat (*4*, *5*, *50*). Conflict and threat theories postulate that ethnic-racial majority members would respond with the strongest threat reaction when diversity is increasing and immigrants are gaining rights (*5*). According to these theories, the threat reaction is based on the perception that ethnic-racial minority groups are endangering the status and power of the majority group in society. Consequently, this threat perception could come from growing proportions of ethnic-racial minority members and would be enhanced when minority members are empowered by inclusive policies, with comparable status and power to that of the majority members. However, we observe that growing diversity spurs threat in unequal sociopolitical contexts, but threat reactions are less likely to materialize in more equal sociopolitical contexts.

The fact that we do not find support for self-selection effects nor for policy responsiveness to immigration attitudes and that we replicate the findings over other contextual characteristics bolsters these theoretical claims. Regarding the self-selection of ethnic-racial majority members, white Americans who are less likely to feel threatened by growing diversity could self-select into more inclusive and diverse environments, including locales with more inclusive immigration policies. Our data do not seem to support that possibility, however. We found that the policy effects hold in studies 1 to 3, taking into account the political partisanship of the self-identified white American participants and the demographic composition of the states and counties. Additional analysis in study 3 (the study with the largest sample) showed that there was no significant mean difference in political orientation (a robust predictor of immigration attitudes) between the participants living in different states (*F*_49,1706_ = 1.016 and *P* = 0.442). Moreover, in study 3, we only found differences between conservative and liberal participants in their emotional reactions but not on other outcomes. Possibly, we did not find the expected interactions with partisanship due to a lack of power. While the sample size in study 3 was based on a priori power analysis, the increasing diversity effect was smaller (*d* = 0.05 across our dependent variables) than foreseen based on previous research (*d* = 0.35 across dependent variables in the studies reviewed). Consequently, we would have needed a larger sample to test the interactions; post-hoc power analysis showed that we would have needed about 4800 participants to detect the main effect of the diversity manipulation across the dependent variables and even more to detect the possible interactions. Similarly, because of lack of power, we could not test the three-way interactions with partisanship and our experimental manipulation and contextual moderators in studies 1 and 2. Regarding the self-selection of immigrants, it could be that immigrants move into environments with more inclusive integration policies. However, the correlation between immigrant inflow and the inclusiveness of policies is not significant in study 4. Migration research also shows that migration policies (that regulate who, and under what conditions, can enter a country for short or long-term settlement) direct migration flows, but integration policies (that regulate the rights and access of immigrants once in the country) are not closely related to the inflow of immigrants (*35*). Regarding policy responsiveness, the longitudinal design in study 4 allowed us to test the direction of causation and showed that policy changes are driving attitudinal changes but not vice versa. Lastly, regarding contextual conditions that could provoke threat reactions, in study 4, we also replicated the policy effect over other possible drivers of threat reactions, such as unemployment rates or the proportion of far-right party seats in parliament. Similarly, in studies 1 and 3, the results hold over the proportion of Democrat seats in state legislature and in study 1, the results hold taking into account whether states lie in the US South or on the US border.

Our findings also have very clear policy implications. Using multiple methods and datasets across continents, we systematically documented that more inclusive policies and changes toward more inclusive policies attenuate or buffer potential threat reactions to increasing ethnic-racial diversity among majorities. Immigration could bolster the labor market and the welfare state in Western countries with aging and shrinking populations. Moreover, ethical and humanitarian considerations call for more immigration. Still, countries often restrict immigration because of political concerns that immigration will provoke tensions and weaken social cohesion. We acknowledge that to implement inclusive policies, policymakers need some level of preexisting political support. Nevertheless, our results show that granting immigrants more equal rights is the way to diffuse tensions and forge social cohesion in ethnically and racially diverse societies.

## MATERIALS AND METHODS

For detailed description of the samples, designs, and measures used in each study, please see the Supplementary Materials.

## Supplementary Materials

### This PDF file includes:

Texts S1 to S4 Figs. S1 to S6 Tables S1 to S5 Legends for tables S6 to S18 References
