# Supplementary material for: Integration policies shape ethnic-racial majorities’ threat reactions to increasing diversity
Source: Sci Adv. 2024 May 29;10(22):eadk8556. doi: 10.1126/sciadv.adk8556 (PMC11135425; doi:10.1126/sciadv.adk8556)
Supplement: Supplementary file 1 — Texts S1 to S4 Figs. S1 to S6 Tables S1 to S5 Legends for tables S6 to S18 References [file sciadv.adk8556_sm.pdf]

Science Advances
AAAS

Supplementary Materials for

Integration policies shape ethnic-racial majorities' threat reactions to increasing diversity

Judit Kende *et al.*

Corresponding author: Judit Kende, j.kende@tilburguniversity.edu

Sci. Adv. **10**, eadk8556 (2024)
DOI: 10.1126/sciadv.adk8556

The PDF file includes:

Texts S1 to S4
Figs. S1 to S6
Tables S1 to S5
Legends for tables S6 to S18
References

Other Supplementary Material for this manuscript includes the following:

Tables S6 to S18

Supplementary Text

Materials and Methods

Supplementary Text 1: Study 1

Participants

There were 469 non-Hispanic white participants, residing in 48 states in the U.S.

Procedure

Levy and Myers collected the data through an online internet survey in June 2020 on a sample fielded by Lucid Labs *(39).*

Experimental Design

The Levy and Myers research included a condition in which participants read about demographics shifts in the United States indicating that white people would soon be becoming a minority with ethnic-racial minority groups becoming the majority and a control condition describing the growth of wild giant panda population *(39).* These two conditions represented a conceptual replication of the two conditions employed in the research by Craig and Richeson on the impact of demographic shifts. These conditions thus were the focus of our current research. Levy and Myers also included three other conditions in their research to explore various elements of demographic changes. In these conditions, participants read that white people would remain the majority due to mixed-race individuals identifying as white (2 conditions with small modifications) or did not speak about the declining numbers of the majority group. Please see the original Levy and Myers paper for detailed description of the procedure. In the Levy and Myers work, non-Hispanic white participants' responses in these three conditions generally did not differ from those of participants in the control condition.

Below is the verbatim information received by participants in the increasing diversity and control condition:
Increasing diversity condition
U.S. Census Bureau Projects whites Will Fall to a Minority in a More Racially Diverse America
By SAMUEL K. RICHARDSON
June 4, 2020
Washington, D.C. – Detailed new projections from the U.S. Census Bureau find that white Americans will fall to a minority of a more racially diverse U.S. population in less than thirty years.
Altogether, between 2020 and 2060, the country's Hispanic and Asian populations will both rise by over 80%, accounting for most of U.S. population growth during that period.
Over the same period, the white population will slow and then begin to shrink. By 2044, whites will, for the first time in U.S. history, be a minority of the population.

Control condition

Panda Population Increases Nearly 17%
By SAMUEL K. RICHARDSON
June 4, 2020
Washington, D.C. – The number of wild giant pandas has increased nearly 17% over the last decade, according to a new survey conducted by the Wildlife Conservation Foundation.

Figures released today show that the global population of wild giant pandas has reached 1,864 – up from 1,596 when their numbers were last surveyed in 2003.

Measures

Emotions

The study used the measurement employed in the American National Election Study, where people are asked sequentially whether they feel or do not feel any of multiple emotions (not towards ethnic-racial minority groups) such as being anxious, proud, angry, hopeful, afraid, excited, happy, depressed, sad, uneasy and disgusted. Principal component analysis showed that the positive and negative emotions load onto two distinct factors, thus we used indices of positive and negative emotions separately.

Policy measures

We used a database of state-level immigrant integration policy data that covered immigration-related legislation enacted in the 50 states from 1990 to 2016. The legislation targeted different groups of immigrants and covered various policy areas such as drivers' licenses, education, family law, healthcare, housing, human trafficking, immigration services, integration (e.g., language services and social services and benefits. We used a measure of positive policies i.e. policies that extended rights to immigrants *(40)* (see policy scores per state in Table S2). Please note that as the raw policy score goes from 14 to 652, in the analysis we divided the policy scores by 638 (i. e. 652-14) so that the policy score now expresses percentage in the range of scores. Thus the range of policy scores in Studies 1 and 3 are comparable to the ranges of the moderators in Study 2 (immigrant percentage) and Study 4 (the theoretical range of the MIPEX scale is from 0 to 100 therefore a 1-point difference is actually the percentage).

Control variables

In each analysis, we controlled for participants' age, gender (male=1, female=2), education (measured as highest completed degree, with higher degree coded as higher score), political partisanship (measured as extremely liberal vs. extremely conservative as a continuous variable, conservative coded as higher score) and for the percentage of white population living in the state based on census data.

Statistical Analysis

We implemented multilevel modelling with participants nested in states using Mplus Version 8 (*51*) (Tables S6 and S7). We compared the condition that discussed "white Americans becoming a minority" to the control condition. We analyzed the effects of these conditions on positive and negative emotions as outcomes in separate models. In each set of analysis, we first ran the null model, followed by the model including the experimental condition, then we added individual

controls and contextual controls. In a final step we included the cross-level interaction between the individual-level experimental effect and the state-level immigrant integration policy.

As robustness checks, we also replicated these final models taking into account the percentage of Democrat seats held in state legislature and including a dummy variable for Southern states and for southern and northern border states (Table S8).

Supplementary Text 2: Study 2

Participants

The final sample included 733 non-Hispanic white participants residing in 37 counties in two U.S. states - New Mexico and Arizona.

Procedure

Huo and colleagues collected data in a telephone survey conducted by ISA Corporation in 2016, please see the original paper for detailed description of the procedure *(16).*

Experimental Manipulation

Participants were randomly assigned to one of two conditions. They were told that lawmakers in their state were considering new policies that are either welcoming of or hostile toward immigrants.
Below is the verbatim information received by participants for the welcoming policy condition (with the information associated with the hostile condition in bold):
Some lawmakers in [Arizona/New Mexico] want the state to adopt policies that would make the state [more/**less**] welcoming for immigrants.
Some policies that have been suggested include:
[adding/**reducing**] more social services for noncitizens
[providing more government documents in languages other than English/**requiring that government documents be available only in English**]
[allowing all residents to get identification cards/**requiring employers to verify the immigration status of their employees**]
In general would you support or oppose these kinds of efforts to make [Arizona/New Mexico] [more/**less**] welcoming for immigrants?

Measures

Positive emotions

After exposure to the experimental stimuli, participants were asked questions about their reactions if their state adopted the proposed set of policies. Would they feel (i) angry, (ii) sad, or (iii) happy? The three items (angry and sad reverse coded, happy) were averaged together to form a single indicator of positive emotions toward the proposal ($\alpha = 0.766$).

Immigrant percentage

We used the 5-year estimates for each county from the 2016 census data reporting the number of foreign-born residents and the total population.

Control variables

In each analysis, we controlled for participants' age, gender (male=1, female=2, other=3), education (measured as highest completed degree, with higher degree coded as higher score), political partisanship (measured as Republican=1, an independent=2, a Democrat=3, or something else=4) and for the percentage of immigrants living in the county.

Statistical Analysis

We implemented multilevel modelling with participants nested in counties using Mplus Version 8 (*51*) (Table S5). We first ran the null model, followed by the model including the experimental condition, then we added individual controls and contextual controls. In a final step we included the cross-level interaction between the individual-level experimental effect and the county-level immigrant percentage.

Supplementary Text 3: Study 3

Study 3 was pre-registered, see preregistration:
https://osf.io/gyatx/?view_only=0fc5acc946034ddab31204dff3c182c8

Participants

The final sample included 1745 white American participants residing in 50 US states.

Procedure

We collected data on Prolific among white residents of the United States. They were invited to participate in an online survey about people's attitudes towards social topics. We obtained informed consent from the participants and participants received compensation for their participation. The research was approved by the IRB at the University of Massachusetts, Amherst and at the Université libre de Bruxelles, Belgium.

Experimental Manipulation

Participants were randomly assigned to one of four conditions. They were assigned to either the increasing diversity (white people becoming a minority) or a control condition. In addition, they were told that lawmakers in the United States were considering new policies that are either welcoming of or hostile toward immigrants.

Below is the verbatim information received by participants for the increasing diversity and welcoming policy condition (Condition 1) and for the control and hostile policy condition Condition 4). The policy manipulation is highlighted in italics here, but it was not highlighted in the experiment itself. The experiment included two other conditions, one combining the same increasing diversity manipulation with the hostile policy condition (Condition 2) and the other combining the control condition with the welcoming policy condition (Condition 3).

"In a Generation, Racial Minorities May Be the U.S. Majority

New U.S. Census Bureau data suggest that America will become a "majority-minority" nation much faster than once predicted. The nation's racial minority population is steadily rising, advancing an unmistakable trend that could make minorities the new American majority by midcentury. The data show a declining number of adults and growing under-18 populations of Hispanics, Asians, and other minorities. Demographers calculate that by 2042, Americans who identify themselves as Hispanic, Black, Asian, American Indian, Native Hawaiian, or Pacific Islander will together outnumber non-Hispanic Whites. The main reasons for the accelerating change are rapid immigration growth and significantly higher birthrates among racial and ethnic minorities. As White baby boomers age past their childbearing years, younger Hispanic parents are having children –and driving U.S. population growth. For example, there are now roughly 9 births for every 1 death among Hispanics, compared to a roughly one-to-one ratio for Whites. The latest figures are predicated on current and historical trends, which can be thrown awry by several variables, including prospective overhauls of public policy.

In parallel, lawmakers in the United States want to adopt policies that would make the country more welcoming for immigrants.

Some policies that would be implemented include: adding more social services for noncitizens, providing more government documents in languages other than English and allowing all residents to get identification cards."

"U.S. Census Bureau Reports Residents Now Move at a Higher Rate

New U.S. Census Bureau data suggest that the rate of geographical mobility, or the number of individuals who have moved within the past year, is increasing. The national mover rate increased from 11.9 percent in 2008 (the lowest rate since the U.S. Census Bureau began tracking the data) to 12.5 percent in 2009. According to the new data, 37.1 million people changed residences in the U.S. within the past year. 84.5 percent of all movers stayed within the same state. Renters were more than five times more likely to move than homeowners. The estimates also reveal that many of the nation's fastest-growing cities are suburbs. Specifically, principal cities within metropolitan areas experienced a net loss of 2.1 million movers, while the suburbs had a net gain of 2.4 million movers. For those who moved to a different county or state, the reasons for moving varied considerably by the length of their move. The latest figures are predicated on current and historical trends, which can be thrown awry by several variables, including prospective overhauls of public policy.

In parallel, lawmakers in the United States want to adopt policies that would make the country less welcoming for immigrants.

Some policies that would be implemented include: reducing more social services for noncitizens, requiring that government documents be available only in English and requiring employers to verify the immigration status of their employees."

Measures

After exposure to the experimental stimuli, participants completed measures of racial status threat, negative emotions, immigration threat perceptions, support for restrictive immigration policies, and attitudes towards undocumented immigrants.

Below is the list of items for each construct.

Negative emotions
Please indicate the extent to which you agree or disagree with each of the following statements (1 to 7).
To what extent does the information make you feel angry at minorities?
To what extent does the information make you feel resentful toward minorities?
To what extent does the information make you feel annoyed at minorities?
To what extent does the information make you feel fearful of minorities?
To what extent does the information make you feel scared of minorities?
To what extent does the information make you feel frightened of minorities?

Racial status threat
Please indicate the extent to which you agree or disagree with each of the following statements (1 to 7).

My racial group should be worried about its place in the future of the U.S.
My racial group should be threatened by growing diversity.
My racial group will benefit from increasing diversity in the U.S. (REV)

Immigration threat perceptions
Please indicate the extent to which you agree or disagree with each of the following statements (1 to 7).
It is generally good for the United States' economy that people come to live here from other countries. (REV)
American cultural life is generally undermined by people coming to live here from other countries.
The United States is made a better place to live by people coming to live here from other countries. (REV)

Support for restrictive immigration policies
Please indicate the extent to which you agree or disagree with each of the following statements (1 to 7).
1.Refugees and asylum-seekers should be allowed into the U.S. (REV)
2.Individuals who do not leave the U.S. after their temporary visa expires should be subject to criminal penalties.
3.The U.S. needs stricter policies for the admission of refugees and asylum seekers into the U.S.
4.Us government funds for refugee programs should be reduced.

Attitudes towards undocumented immigrants
Using the "thermometer," please indicate how you feel about the group listed.
Undocumented immigrants
Answers on sliding scale anchored by 1 = very cold and 100 = very warm.

Policy measures

We used the same database of state-level immigrant integration policy as in Study 1 *(34)*.

Control variables

In each analysis, we controlled for participants' age, gender (2.00=male, 3.00=female, 4.00=other), education (measured as highest completed degree, with higher degree coded as higher score), political partisanship (measured as extremely liberal vs. extremely conservative as a continuous variable, conservative coded as higher score) and for the percentage of white people living in the state.

Statistical Analysis

We implemented multilevel modelling with participants nested in states using Mplus Version 8 (*51*) (Tables S6 to S10). We analyzed the effects of the experimental conditions on racial status threat, negative emotions, immigration threat perceptions, support for restrictive immigration policies and attitudes towards undocumented immigrants as outcomes in separate models. In each set of analysis, we first ran the null model, followed by the model including the increasing diversity manipulation and individual controls, then the model including the policy manipulation and individual controls. Afterwards we tested the 2-way interaction between the increasing diversity manipulation and the policy manipulation. In a final step we included the 3-way cross-level interaction between the individual-level experimental effects (increasing diversity and policy) and the state-level immigrant integration policy.

To test the robustness of the effects, we also replicated the final interaction model controlling for the percentage of seats held by Democrats in the state House and Senate (Table S15).

Supplementary Text 4: Study 4

Participants

The sample consisted of 499.075 participants who identify as citizens or nationals of their countries of residence. We used a longitudinal dataset covering the years 2007 to 2017 compiled by Claassen and McLaren, who harmonized survey responses on immigration from nationally representative surveys across Europe *(13).* We used data from only those countries where survey responses were available for at least 3 years and where we could match the survey responses to the Migrant Integration Policy Index (MIPEX) *(42)*. The data that we used was gathered in 385 nationally representative surveys in six survey projects: the Eurobarometer, European Social Survey, European Values Study, World Values Survey, Pew Global Attitudes Survey and the International Social Survey Programme. The surveys covered the following countries: Austria, Belgium, Croatia, Cyprus, Czech Republic, Denmark, Estonia, Finland, France, Germany, Greece, Hungary, Iceland, Ireland, Italy, Latvia, Lithuania, Luxembourg, Malta, Netherlands, Norway, Poland, Portugal, Romania, Slovakia, Slovenia, Spain, Sweden, Switzerland, and the United Kingdom.

Measures

Immigration attitudes were calculated from all available items on immigration across surveys in a given year in a country by Claassen and McLaren *(13)*. Items on immigration included the perception of immigrants, perception of immigration and immigration policy preferences. Example items include “Is [country] made a worse or a better place to live by people coming to live here from other countries?” “In your opinion, should we allow more immigrants to move to our country, fewer immigrants, or about the same as we do now? “.

Immigrant integration policies

The MIPEX is the most comprehensive and detailed index of immigrant integration policies in Europe. More specifically, it is a country-level index of immigrant integration policies that simultaneously considers 167 policy indicators from eight policy domains (i.e., health care, education, political participation, labor market, antidiscrimination laws, permanent residence, access to nationality, family reunion). The indicators measure whether immigrants have comparable rights and access to services in these policy domains vis-à-vis natives. The index is based on expert surveys in each country and has been updated yearly since 2007. Furthermore, overall it forms a reliable scale (*52*). For these reasons, it has been widely used in comparative sociological and social psychological research *(20).*

Immigrant inflow

Data for immigrant inflows were compiled from three sources by Claassen and McLaren *(13)*: the Organization for Economic Co-operation and Development (OECD), Eurostat, and the Determinants of International Migration (DEMIG) project. Immigrant inflow is measured as the percentage of the total population in each country and year.

Statistical Analysis

To test our hypotheses, we employed a multilevel procedure for analyzing cross-sectional time-series data (*53, 11-12*). This model specification allows to test simultaneously a cross-sectional effect comparing different countries and a longitudinal effect investigating change within countries. First, we calculated two predictors to test the cross-sectional effect and compare counties. We calculated these predictors by taking the mean of immigrant presence and the mean of policies across all available years for each country. Second, we computed two longitudinal predictors to capture the effect of change within each country. For these predictors, we subtracted the year-specific immigrant inflow and polices in each country from the mean of immigrant inflow and policies across all available years for each country. Thus, with such specification, it is possible to disaggregate immigrant presence and policies into a between-country coefficient and a within-country coefficient. Following previous research on diversity *(11-12),* we use the between-country cross-sectional coefficient to assess the long-term effects of immigrant inflow and policies and the within-country change coefficient to measure the short-term effects of yearly increases in immigrant inflow and changes in policies. We fitted a two-level multilevel model where surveys in specific years were nested within countries (Table S12). We first ran the null model, followed by the model including the main effects of immigrant inflow, then the main effects of immigrant inflow (both between-country and within-country

coefficient), then the main effects of immigrant integration policy (both between-country and within-country coefficient). Finally, we included the interaction between yearly change in immigrant inflow and yearly change in policies.

Afterwards, to test the robustness of the interaction, we included in the final interaction model other country-level factors that could potentially exacerbate attitudes such as national wealth (natural log of GDP per capita, based on merged Maddison, World Development Indicators and IMF measures), unemployment rates, income inequality (GINI index), proportion of immigrants, and proportion of far-right seats in parliament (Table S13). We also tested a reverse causation model predicting yearly change in policies (country year level) and average policies (country level) from the main effects of immigrant inflow and immigration (Table S14).

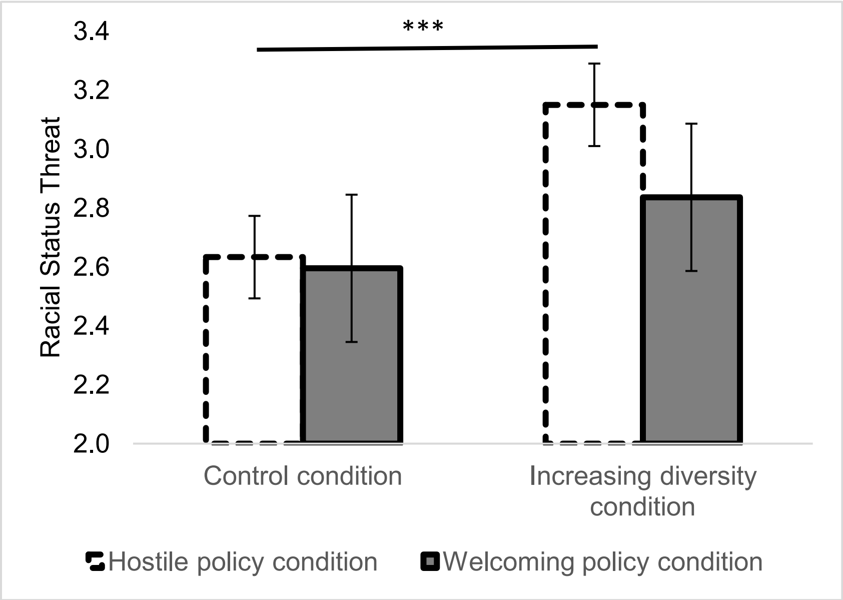

Fig. S1.

Study 3. *Increasing diversity manipulation* (dichotomous variable) and *policy manipulation* (dichotomous variable) predicting *racial status threat* in states with *more inclusive policies* (continuous variable, defined as +2 SD from the mean). Error bars represent standard errors.

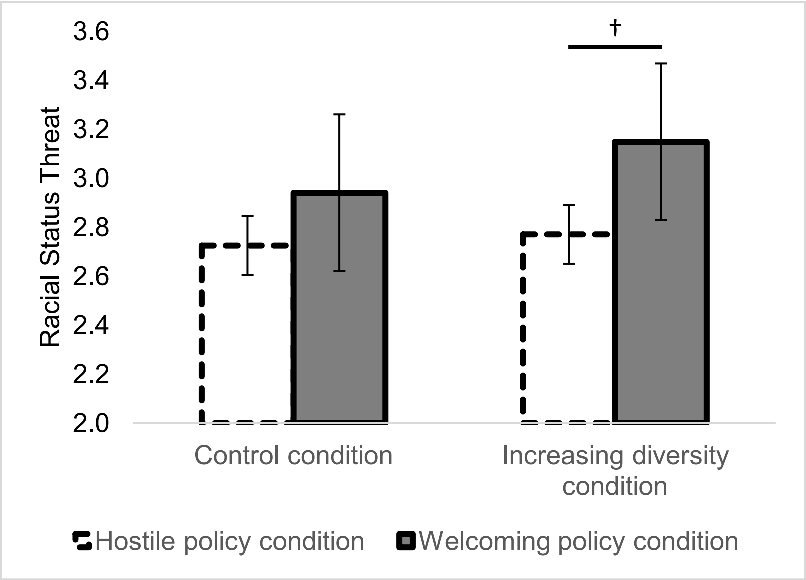

Fig. S2.

Study 3. *Increasing diversity manipulation* (dichotomous variable) and *policy manipulation* (dichotomous variable) predicting *racial status threat* in states with *more exclusive policies* (continuous variable, defined as +2 SD from the mean). Error bars represent standard errors.

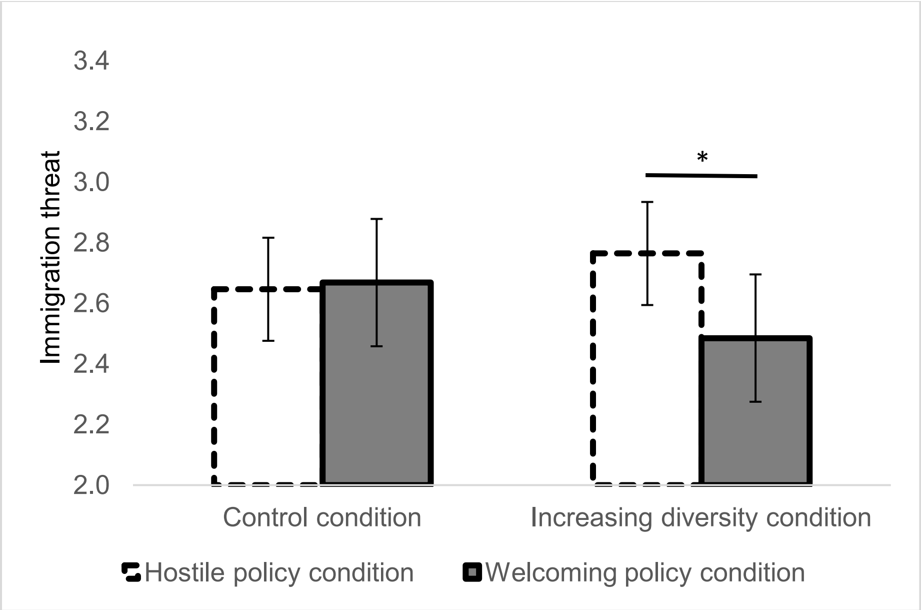

Fig. S3.

Study 3. *Increasing diversity* manipulation (dichotomous variable) and *policy manipulation* (dichotomous variable) predicting *perceptions of immigration threat* in states with *more inclusive policies* (continuous variable, defined as +2 SD from the mean). Error bars represent standard errors.

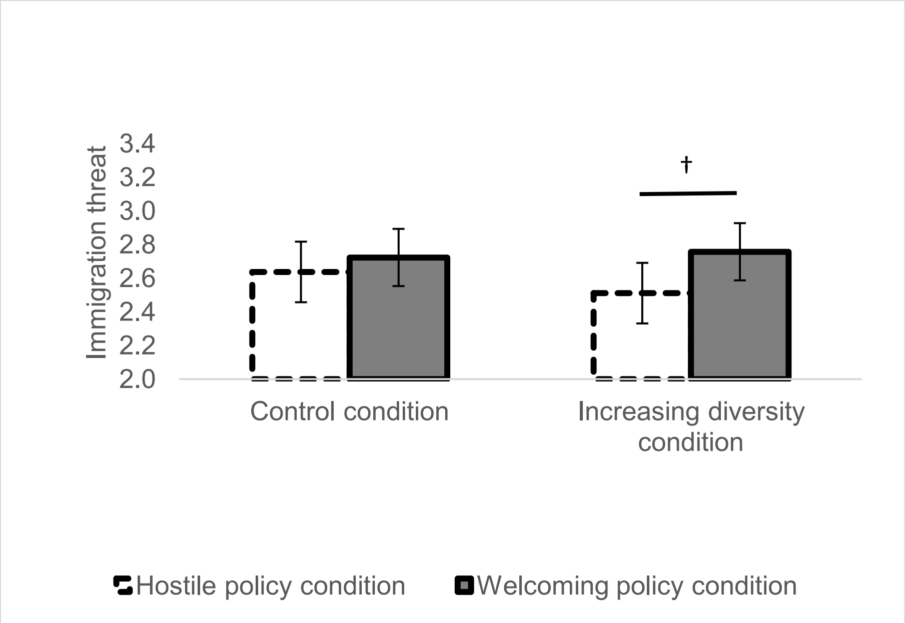

Fig. S4.

Study 3. *Increasing diversity* manipulation (dichotomous variable) and *policy manipulation* (dichotomous variable) predicting *perceptions of immigration threat* in states with *more exclusive policies* (continuous variable, defined as +2 SD from the mean). Error bars represent standard errors.

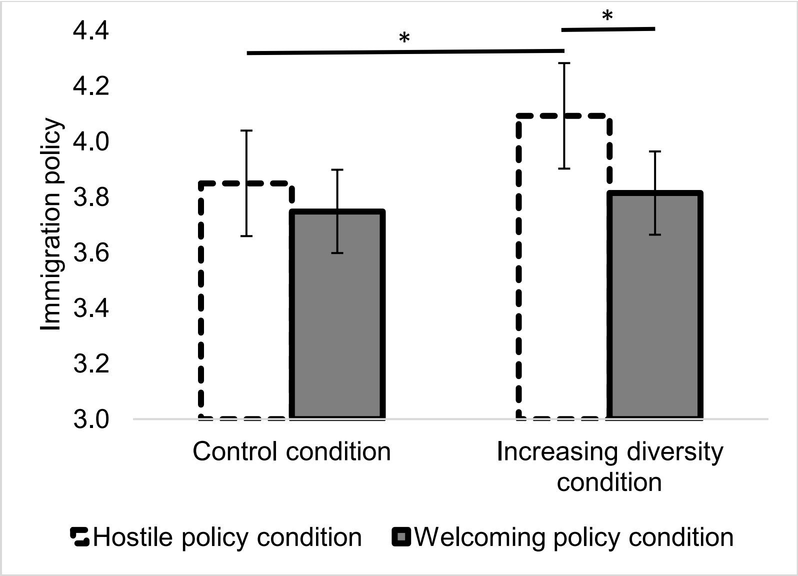

Fig. S5.

Study 3. *Increasing diversity* manipulation (dichotomous variable) and *policy manipulation* (dichotomous variable) predicting *support for restrictive immigration policies* in states with *more inclusive policies* (continuous variable , defined as +2 SD from the mean). Error bars represent standard errors.

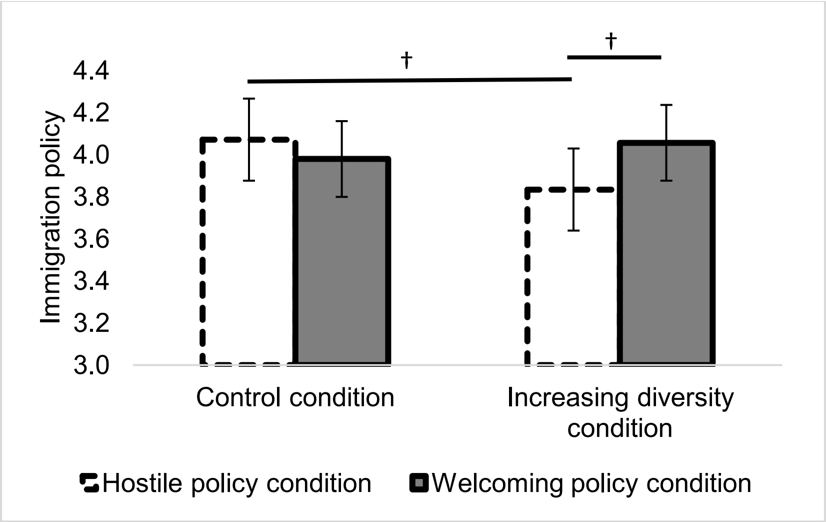

Fig. S6.

Study 3. *Increasing diversity* manipulation (dichotomous variable) and *policy manipulation* (dichotomous variable) predicting *support for restrictive immigration policies* in states with *more exclusive policies* (continuous variable, defined as +2 SD from the mean. Error bars represent standard errors.

Table S1.
Study 1 descriptives.

	Mean	SD	Range
**Predictor**			
Integration Policies	120.90	102.64	14.65 to 652.12
**Control variables**			
Age	44.00	17.00	18 to 88
Gender	49% male		
Education	43 % college degree		1 to 8
Political partisanship	5.47	3.51	1 to 10
white % in state	0.76	0.14	0.247 to 0.953
**Outcome variables**			
Positive emotions	0.46	0.43	0 to 1
Negative emotions	0.15	0.28	0 to 1

Table S2.

Study 1 and 3. Index of weighted positive immigrant integration policies by state *(34)*

State	Number of positive policies (weighted)	State	Number of positive policies (weighted)
Alabama	78,11	Montana	60,66
Alaska	18,59	Nebraska	95,41
Arizona	209,92	Nevada	114,82
Arkansas	139,51	New Hampshire	56,72
California	652,12	New Jersey	116,28
Colorado	266,03	New Mexico	113,01
Connecticut	173,81	New York	247,47
Delaware	85,39	North Carolina	63,38
Florida	185,99	North Dakota	43,32
Georgia	123,09	Ohio	59,12
Hawaii	100,18	Oklahoma	57,09
Idaho	72,62	Oregon	102,24
Illinois	299,05	Pennsylvania	33,82
Indiana	88,36	Rhode Island	154,20
Iowa	47,79	South Carolina	101,38
Kansas	53,71	South Dakota	38,62
Kentucky	33,82	Tennessee	107,23
Louisiana	109,86	Texas	179,46
Maine	126,50	Utah	151,15
Maryland	136,88	Vermont	63,17
Massachusetts	46,62	Virginia	271,36
Michigan	100,34	Washington	132,50
Minnesota	113,59	West Virginia	61,65
Mississippi	40,13	Wisconsin	42,99
Missouri	42,11	Wyoming	14,65

Table S3.
Study 2 descriptives.

	Mean	SD	Range
**Predictor**			
Immigrant percentage	11.4	0.04	2 to 33
**Control variables**			
Age	60.269	17.52	18 to 96
Gender	45 % male		
Education	46 % college degree		
Political partisanship	31 % Republican		
**Outcome variables**			
Positive emotions	2.78	0.95	1 to 4

Table S4.
Study 3 descriptives.

	Mean	SD	Range
**Predictor**			
Integration Policies	120.9	102.64	14.65 to 652.12
**Control variables**			
Age	41.88	13.61	18 to 93
Gender	49% male		
Education	68 % college degree		2 to 7
Political partisanship	4.29	1.68	2 to 8
white % in state	0.75	0.09	0.247 to 0.953
**Outcome variables**			
Racial status threat	2.78	1.52	1 to 7
Negative emotions	1.79	1.16	1 to 7
Immigration threat perceptions	3.53	1.61	1 to 7
Support for restrictive immigration polices	2.69	1.43	1 to 7
Attitudes towards undocumented immigrants	5.71	2.89	1 to 10

Table S5.
Study 4 descriptives.

	Mean	SD	Range
**Predictors**			
Immigrant inflow	0.70	0.61	0.09 to 3.11
Immigrant integration policies	54.15	14.14	32.44 to 87.67
**Control variables**			
National wealth	10.47	0.36	9.75 to 11.50
Unemployment rates	0.09	0.05	0.02 to 0.27
Income inequality	0.29	0.03	0.23 to 0.36
Proportion of immigrants	8.72	8.58	0.14 to 47.20
Proportion of far-right seats in parliament	0.10	0.16	0 to 0.80
**Outcome variables**			
Immigration attitudes	0.41	1.21	-2.30 to 3.31

Supplementary Tables file contains

Table S6.
Study 1. Multilevel models predicting negative emotions from the interaction between increasing diversity manipulation and state immigrant integration policies.

Table S7.
Study 1. Stepwise models predicting positive emotions from the interaction between increasing diversity manipulation and state immigrant integration policies

Table S8.
Study 1. Robustness checks: Final interaction models (increasing diversity X policy) including proportion of Democrat seats in state House and Senate, dummy variable for Southern states and for southern or northern border states

Table S9.
Study 2. Stepwise models predicting positive emotions from the interaction between policy manipulation and county immigrant percentage

Table S10.
Study 3. Stepwise models predicting racial status threat from the interaction between increasing diversity manipulation, policy manipulation and state immigrant integration policies

Table S11.
Study 3. Stepwise models predicting negative emotions from the interaction between increasing diversity manipulation, policy manipulation and state immigrant integration policies

Table S12.
Study 3. Stepwise models predicting immigration threat perceptions from the interaction between increasing diversity manipulation, policy manipulation and state immigrant integration policies

Table S13.
Study 3. Stepwise models predicting support for restrictive immigration policy from the interaction between increasing diversity manipulation, policy manipulation and state immigrant integration policies

Table S14.
Study 3. Stepwise models predicting attitudes towards undocumented immigrants (higher score more positive attitudes) from the interaction between increasing diversity manipulation, policy manipulation and state immigrant integration policies

Table S15.
Study 3. Robustness checks: Final interaction models including proportion of Democrat seats in state House and Senate

Table S16.
Study 4. Stepwise models predicting immigration attitudes from immigration inflow and immigrant integration policy (mean across years and yearly change) and their interaction

Table S17.
Study 4. Robustness checks. Stepwise models predicting immigration attitudes from the interaction of immigration inflow change and immigrant integration policy change over additional contextual predictors (GDP, unemployment rates, GINI, immigrants stock and percentage of far-right party seats in parliament).

Table S18.
Study 4. Reverse causation model. Model predicting yearly change in policies (country-year level) and average policies (country level) from the main effects of immigrant inflow and immigration.

REFERENCES AND NOTES

1. R. D. Putnam, *E Pluribus Unum*: Diversity and community in the twenty-first century the 2006 Johan Skytte Prize Lecture. *Scand. Polit. Stud.* **30**, 137–174 (2007).

2. J. Hainmueller, D. J. Hopkins, Public attitudes toward immigration. *Annu. Rev. Polit. Sci.* **17**, 225–249 (2014).

3. J. A. Richeson, S. R. Sommers, Toward a social psychology of race and race relations for the twenty-first century. *Annu. Rev. Psychol.* **67**, 439–463 (2016).

4. L. D. Bobo, Prejudice as group position: Microfoundations of a sociological approach to racism and race relations. *J. Soc. Issues* **55**, 445–472 (1999).

5. W. G. Stephan, O. Ybarra, K. Rios, in *Handbook of prejudice, stereotyping, and discrimination, 2nd ed.*, T. D. Nelson, Ed. (Psychology Press, New York, NY, US, 2016), pp. 255–278.

6. B. Meuleman, E. Davidov, J. Billiet, Changing attitudes toward immigration in Europe, 2002–2007: A dynamic group conflict theory approach. *Soc. Sci. Res.* **38**, 352–365 (2009).

7. M. A. Craig, J. M. Rucker, J. A. Richeson, Racial and political dynamics of an approaching “majority-minority” United States. *Ann. Am. Acad. Pol. Soc. Sci.* **677**, 204–214 (2018).

8. E. Kaufmann, M. J. Goodwin, The diversity Wave: A meta-analysis of the native-born white response to ethnic diversity. *Soc. Sci. Res.* **76**, 120–131 (2018).

9. M. A. Craig, J. A. Richeson, On the precipice of a “majority-minority” America: Perceived status threat from the racial demographic shift affects white americans’ political ideology. *Psychol. Sci.* **25**, 1189–1197 (2014).

10. M. A. Craig, J. M. Rucker, J. A. Richeson, The pitfalls and promise of increasing racial diversity: Threat, contact, and race relations in the 21st century. *Curr. Dir. Psychol. Sci.* **27**, 188–193 (2017).

11. M. R. Ramos, M. R. Bennett, D. S. Massey, M. Hewstone, Humans adapt to social diversity over time. *Proc. Natl. Acad. Sci. U.S.A.* **116**, 12244–12249 (2019).

12. M. R. Ramos, S. Schumann, M. Hewstone, The role of short-term and longer term immigration trends on voting for populist radical right parties in Europe. *Soc. Psychol. Pers. Sci.*. **13**, 816–826 (2022).

13. C. Claassen, L. McLaren, Does immigration produce a public backlash or public acceptance? Time-series, cross-sectional evidence from thirty european democracies. *Brit. J Polit Sci.* **52**, 1013–1031 (2022).

14. E. Schlueter, B. Meuleman, E. Davidov, Immigrant integration policies and perceived group threat: A multilevel study of 27 Western and Eastern European countries. *Soc. Sci. Res.* **42**, 670–682 (2013).

15. S. Guimond, R.J. Crisp, P. de Oliveira, R. Kamiejski, N. Kteily, B. Kuepper, R.N. Lalonde, S. Levin, F. Pratto, F. Tougas, J. Sidanius, A. Zick, Diversity policy, social dominance, and intergroup relations: Predicting prejudice in changing social and political contexts. *J. Pers. Soc. Psychol.* **104**, 941–958 (2013).

16. Y. J. Huo, J. F. Dovidio, T. R. Jiménez, D. J. Schildkraut, Local policy proposals can bridge Latino and (most) white Americans' response to immigration. *Proc. Natl. Acad. Sci. U.S.A.* **115**, 945–950 (2018).

17. J. Kende, O. Sarrasin, A. Manatschal, K. Phalet, E. G. T. Green, Policies and prejudice: Integration policies moderate the link between immigrant presence and anti-immigrant prejudice. *J. Pers. Soc. Psychol.* **123**, 337–352 (2022).

18. M. E. Tankard, E. L. Paluck, Norm perception as a vehicle for social change. *Soc. Issues Policy Rev.* **10**, 181–211 (2016).

19. E. G. T. Green, C. Staerklé, in *The Oxford handbook of political psychology, 3rd ed.* (Oxford University Press, New York, NY, US, 2023), pp. 1016–1061.

20. G. Solano, S. Yilmaz, T. Huddleston, The link between migration policies and migration and migrant integration dynamics. *Leuven, Belgium: HumMingBird Project*, (2022).

21. M. Wright, I. Bloemraad, Is there a trade-off between multiculturalism and socio-political integration? Policy regimes and immigrant incorporation in comparative perspective. *Perspect. Polit.* **10**, 77–95 (2012).

22. M. Marbach, J. Hainmueller, D. Hangartner, The long-term impact of employment bans on the economic integration of refugees. *Sci. Adv.* **4**, eaap9519 (2023).

23. A. Hager, H. Hilbig, S. Riaz, Refugee labor market access increases support for immigration. *Comp. Pol. Stud.* **57**, 749–777 (2023).

24. E. G. T. Green, E. P. Visintin, O. Sarrasin, M. Hewstone, When integration policies shape the impact of intergroup contact on threat perceptions: A multilevel study across 20 European countries. *J. Ethn. Migr. Stud.* **46**, 631–648 (2020).

25. J. Kende, K. Phalet, W. Van den Noortgate, A. Kara, R. Fischer, Equality revisited: A cultural meta-analysis of intergroup contact and prejudice. *Soc. Psychol. Pers. Sci.* **9**, 887–895 (2018).

26. A. A. Seate, D. Mastro, Media's influence on immigration attitudes: An intergroup threat theory approach. *Commun. Monogr.* **83**, 194–213 (2016).

27. D. Myers, M. Levy, Racial population projections and reactions to alternative news accounts of growing diversity. *Ann. Am. Acad. Pol. Soc. Sci.* **677**, 215–228 (2018).

28. N. Foner, P. Simon, *Fear, anxiety, and national identity: Immigration and belonging in North America and Western Europe*. (Russell Sage Foundation, 2015).

29. M. Safi, *Migration and Inequality*. (Polity Books, 2020), 99, e1, e3.

30. S. O. Roberts, M. T. Rizzo, The psychology of American racism. *Am. Psychol.* **76**, 475–487 (2021).

31. G. Asante, S. Sekimoto, C. Brown, Becoming “Black”: Exploring the racialized experiences of African immigrants in the United States. *Howard J. Commun.* **27**, 367–384 (2016).

32. R. Sáenz, K. Manges Douglas, A call for the racialization of immigration studies: On the transition of ethnic immigrants to racialized immigrants. *Sociol. Race Ethnicity* **1**, 166–180 (2015).

33. H. De Haas, S. Castles, M. J. Miller, *The age of migration: International population movements in the modern world*. (Bloomsbury Publishing, London, 2019).

34. P. A. Silverstein, Immigrant racialization and the new savage slot: Race, migration, and immigration in the new Europe. *Ann. Rev. Anthropol.* **34**, 363–384 (2005).

35. H. de Haas, M. Czaika, M.L. Flahaux, E. Mahendra, K. Natter, S. Vezzoli, M. Villares-Varela, International migration: Trends, determinants, and policy effects. *Popul. Dev. Rev.* **45**, 885–922 (2019).

36. M. Ambrosini, A. van Hootegem, P. Bevelander, P. Daphi, E. Diels, T. Fouskas, A. Hellström, S. Hinger, A. Hondeghem, A. Kováts, A. Mazolla, E. Mescoli, B. Meuleman, A. Rea, M. Reidsma, A. Roblain, V. Stern, *The refugee reception crisis: Polarized opinions and mobilizations*. (Éditions de l'Université de Bruxelles, Brussels, 2019).

37. L. Buonanno, in *The European Union in Crisis*, D. Dinan, N. Nugent, W. E. Paterson, Eds. (Palgrave Macmillan, London, 2017), pp. 100–130.

38. B. M. Riek, E. W. Mania, S. L. Gaertner, Intergroup threat and outgroup attitudes: A meta-analytic review. *Pers. Soc. Psychol. Rev.* **10**, 336–353 (2006).

39. M. Levy, D. Myers, Racial projections in perspective: Public reactions to narratives about rising diversity. *Perspect. Polit.* **19**, 1147–1164 (2021).

40. A. Filindra, S. Pearson-Merkowitz, *Database of state-level immigration policy bills and enactments*. (Chicago: University of Illinois at Chicago, 2016).

41. B. A. Bell, G. B. Morgan, J. A. Schoeneberger, J. D. Kromrey, J. M. Ferron, How low can you go? *Methodology* **10**, 1–11 (2014).

42. G. Solano, T. Huddleston, "Migrant Integration Policy Index 2020," (2020).

43. L. Platt, J. Polavieja, J. Radl, Which integration policies work? The heterogeneous impact of national institutions on immigrants' labor market attainment in Europe. *Int. Migr. Rev.* **56**, 344–375 (2022).

44. E. L. Paluck, S. A. Green, D. P. Green, The contact hypothesis re-evaluated. *Behav. Public Policy* **3**, 129–158 (2019).

45. E. G. T. Green, O. Sarrasin, in *The Routledge Companion to Migration, Communication, and Politics*, S. M. Croucher, J. R. Caetano, E. A. Campbell, Eds. (Routledge/Taylor & Francis Group, 2018), pp. 282–295.

46. M. Kauff, F. Asbrock, S. Thörner, U. Wagner, Side effects of multiculturalism. *Pers. Soc. Psychol. Bull.* **39**, 305–320 (2013).

47. K. Dhont, A. Van Hiel, We must not be enemies: Interracial contact and the reduction of prejudice among authoritarians. *Personal. Individ. Differ.* **46**, 172–177 (2009).

48. S. Stewart, R. Willer, The effects of racial status threat on White Americans' support for Donald Trump: Results of five experimental tests. *Group Process. Intergroup Relat.* **25**, 791–810 (2022).

49. X. Brown, J. M. Rucker, J. A. Richeson, Political ideology moderates White Americans' reactions to racial demographic change. *Group Process. Intergroup Relat.* **25**, 642–660 (2022).

50. L. Quillian, Prejudice as a response to perceived group threat: Population composition and anti-immigrant and racial prejudice in Europe. *Am. Sociol. Rev.* **60**, 586–611 (1995).

51. L. K. Muthén, B. Muthén, *Mplus User's Guide. Eighth Edition*. (Muthén & Muthén, Los Angeles, CA, 2017).

52. D. Ruedin, The reliability of MIPEX indicators as scales (2011).

53. M. Fairbrother, Two multilevel modeling techniques for analyzing comparative longitudinal survey datasets. *Polit. Sci. Res. Methods* **2**, 119–140 (2014).
